# Influence of Radiation Sterilization on Properties of Biodegradable Lactide/Glycolide/Trimethylene Carbonate and Lactide/Glycolide/ε-caprolactone Porous Scaffolds with Shape Memory Behavior

**DOI:** 10.3390/ma9010064

**Published:** 2016-01-20

**Authors:** Piotr Rychter, Natalia Śmigiel-Gac, Elżbieta Pamuła, Anna Smola-Dmochowska, Henryk Janeczek, Wojciech Prochwicz, Piotr Dobrzyński

**Affiliations:** 1 Faculty of Mathematics and Natural Sciences, Jan Dlugosz University in Czestochowa, Armii Krajowej 13/15 Av., Czestochowa 42-218, Poland; p.rychter@ajd.czest.pl (P.R.); nsgac@cmpw-pan.edu.pl (N.Ś.-G.); w.prochwicz@ajd.czest.pl (W.P.); 2 Centre of Polymer and Carbon Materials, Polish Academy of Sciences, Curie-Sklodowska 34 St., Zabrze 41-800, Poland; asmola@cmpw-pan.edu.pl (A.S.-D.); hjaneczek@cmpw-pan.edu.pl (H.J.); 3 Department of Biomaterials, Faculty of Materials Science and Ceramics, AGH University of Science and Technology, Al Mickiewicza 30, Kraków 30-059, Poland; epamula@agh.edu.pl

**Keywords:** biodegradable polymer, gamma sterilization, electron beam sterilization, radiation degradation, scaffold, shape memory

## Abstract

The aim of the study was the evaluation of gamma irradiation and electron beams for sterilization of porous scaffolds with shape memory behavior obtained from biodegradable terpolymers: poly(l-lactide-*co*-glycolide-*co*-trimethylene carbonate) and poly(l-lactide-*co*-glycolide-*co*-ɛ-caprolactone). The impact of mentioned sterilization techniques on the structure of the scaffolds before and after the sterilization process using irradiation doses ranged from 10 to 25 kGy has been investigated. Treatment of the samples with gamma irradiation at 15 kGy dose resulted in considerable drop in glass transition temperature (T_g_) and number average molecular weight (M_n_). For comparison, after irradiation of the samples using an electron beam with the same dose, no significant changes in structure or properties of examined scaffolds have been noticed. Higher doses of irradiation via electron beam caused essential changes of the scaffolds’ pores resulting in partial melting of their surface. Nevertheless, obtained results have revealed that sterilization with electron beam, when compared to gamma irradiation, is a better method because it does not affect significantly the physicochemical properties of the scaffolds. Both used methods of sterilization did not influence the shape memory behavior of the examined materials.

## 1. Introduction

One of the criteria which must to be met for biomaterials is the possibility of their sterilization leading to destruction of pathogenic microorganisms. For a majority of biomedical devices, sterilization is mandatory and a validation before their clinical use is necessary. However, in the case of materials prepared from biodegradable polyesters or polyester-carbonates possessing susceptibility to hydrolysis and low thermal resistance, fulfilling these criteria is challenging.

Even when low-temperature methods for sterilization processes are being used, influence of this treatment later results in changes of final physical properties of this material and, consequently, on its application [1,2]. With this respect, it is crucial to select appropriate methods of sterilization to avoid any undesirable damages of material.

Since, the three-dimensional (3D) scaffolds are designed for cell growth within their entire volume, they possess unique properties, such as relatively poor mechanical properties, very high porosity, and specific surface area, which provide the optimal condition for growing cells [3,4,5]. Proper choice of a sterilization method is especially difficult when the examined material must keep its thermally-induced shape memory properties.

Biomaterials of this type are capable of returning from a temporary to a previously-programmed shape as a result of a mechanical response to thermal stimulation at a temperature close to body temperature [6,7,8]. This means that scaffolds with temporary shape, which was accomplished via two steps (first—compression at higher temperature; second—rapid cooling at low temperature) must be sterilized in such a way as to avoid too fast a relaxation of this material to its permanent (initial) shape. With this respect, the sterilization should proceed as fast as possible, at the lowest possible temperature, much lower than the temperature inducing the return of the sample to its permanent shape.

These limitations result in a narrow choice of sterilization methods. To obtain the required decontamination sterilization by ethylene oxide (EtO) should be undertaken at temperatures between 45 and 60 °C, which is not suitable for the examined scaffolds [9].

According to literature data, the vast majority of polymeric materials with shape memory behavior have been sterilized using a gas plasma sterilization process at room temperature [5,10,11]. However, in the case of this method, when used for sterilization of porous scaffolds, the increase in pore interconnection has been observed. This phenomenon may be explained by the fact that foams have been exposed to variable pressure during the sterilization cycle (from atmospheric to 54–67 Pa) that should have promoted a collapse of thinner pore walls [11]. Additionally, the method of sterilization should assure sterility in the entire volume of the material which is not always possible to achieve during plasma or chemical sterilization of highly porous scaffolds.

Following the literature, the most popular sterilization method of porous scaffolds exhibiting shape memory behavior is immersion of the samples in 70% ethanol (*v*/*v* in water) followed by UV irradiation (*λ* = 254 nm) for 10 min [12,13]. However, this method does not provide total decontamination and is classiﬁed rather as a disinfection, than sterilization, because of its inability to destroy hydrophilic viruses or bacterial spores [14].

Based on the already existing reports related to sterilization of highly porous scaffolds with shape memory properties dedicated for the cell growth purposes, the use of radiation methods seems to be the most effective. Radiation sterilization, among other advantages like short time of treatment, proceeded at room temperature, or lack of toxic residues after sterilization, also possess high decontamination efficiency even for materials which are difficult for internal penetration of the sterilization agent, like porous scaffolds.

Current investigations devoted to optimization of sterilization methods of polymeric scaffolds or nanofibers demonstrate ambiguous and contradictory results. It may be explained by the fact, that the scaffolds’ composition and microstructure of the used polymer chain are key factors inﬂuencing the degradation reactions occurring upon irradiation [15]. Sterilization of scaffolds made from poly(ε-caprolactone) using gamma irradiation caused an essential decrease in average molecular weights and glass transition temperature, leading to deterioration in their mechanical properties. However, it is worth noting that the mentioned treatment did not influence cell attachment and growth [3].

It has also been shown [15] that a copolymer containing dioxepanone (DXO) units and a high amount of l-lactide was more prone to degradation by chain-scission than a material containing higher amount of caproyl units. Usefulness of radiation methods to sterilize porous materials obtained from polylactide and poly(trimethylene carbonate) blends has been also proved, despite the fact of lowering their glass transition, molecular weight, and the deterioration of thermo-mechanical properties [16].

Comparative studies of the type of sterilization method on the properties of acrylate-based copolymers exhibiting shape memory behavior have revealed better usefulness of gamma irradiation as compared to sterilization via low temperature plasma techniques. The acrylic networks sterilized by plasma elicited a strong cytotoxic response [16]. The influence of applied sterilization methods on the biodegradable materials is dependent both on the structure and composition of polymeric materials but also on the scaffold’s morphology.

The aim of this study is examination of the usefulness of radiation methods for the sterilization of scaffolds made from terpolymers l-lacide/glycolide/ε-caprolactone and l-lactide/glycolide/TMC (trimethylene carbonate) exhibiting shape memory behavior. Results related to effectiveness of proposed scaffolds in minimally invasive surgery for bone defects treatment have already been published by us [17,18].

## 2. Results and Discussion

### 2.1. Scaffolds Formulation and Procedure of Investigations

In this study porous scaffolds with shape memory behavior designed for large bone defects treatment via minimally invasive surgery approach were tested. The scaffolds were formed using two different types of microblock terpolymers obtained via ring opening polymerization (ROP): poly(l-lactide-co-glycolide-co-ε-caprolactone) LA/GL/CL and poly(l-lactide-co-glycolide-trimethylene carbonate) LA/GL/TMC with similar molecular weight and polydispersity.

**Scheme 1:**
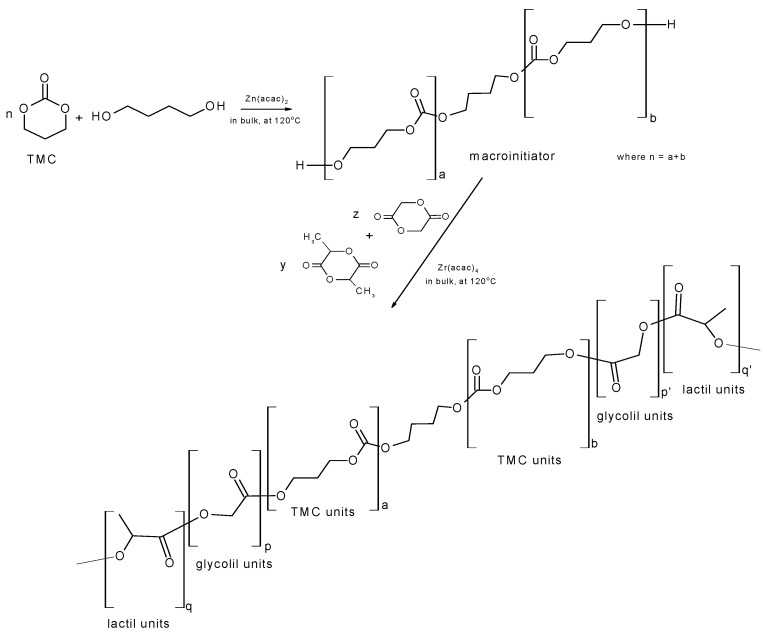
The two-step synthesis procedure of l-lactide/glycolide/trimethylene carbonate terpolymers (LGT21 and LGT40).

The first type of terpolymers has been obtained in two stage reaction (Scheme 1). At the first stage, the hydroxyl-terminated oligocarbonate shave been synthesized in order to be used afterward, as a macroinitiator for the synthesis of terpolymer LA/GL/TMC. Obtained terpolymer was built of lactidyl (LL) and carbonate microblocks (TT) bonded mainly by short lactidyl/glicolidyl (GGLL, LLGG) and glycolidyl (GG) sequences) [8], (see also NMR spectra, Supplementary Materials, Figures S1 and S2).

**Scheme 2:**
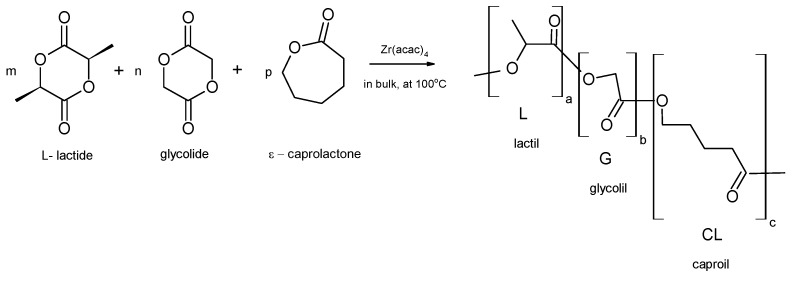
l-lactide/glycolide/caprolactoneterpolymer (LGC) synthesis.

The third copolymer (LGC) has been obtained during one stage reaction via ROP of l-lactide, glycolide and ε-caprolactone (Scheme 2) proceeded in bulk and initiated with zirconium(IV) acetylacetonate (Zr(acac)_4_). The chain of the synthesized terpolymer consisted mainly of longer lactidyl LLLL microblocks with embedded shorter glicolidyl/caproil and glycolil/caproil sequences (CLGGCL, CLGGGCL, CLGCL) [19], (see also NMR spectra, Supplement Materials, Figure S3).

In addition to the scaffolds containing caproil units LA/GL/CL (Table 1, LGC), two types of scaffolds consisting of different amount of carbonate units in polymer chains have been treated with radiation sterilization (Table 1, LGT50 and LGT20). A detailed description of the polymer’s microstructure, as well as methods of its determination allowing it to obtain optimal properties for shape memory effect have been already reported [7,8,19].

Scaffolds were produced via solvent casting/particulate leaching technique using the same porogen (sodium chloride particles) [17,20]. Obtained scaffolds had a similar average porosity and diameter of pores. Principal properties of the used terpolymers, as well as scaffolds made from them have been presented in Table 1.

To obtain a temporary shape of the scaffolds, cylindrically shaped materials with diameter *ca*. 10 mm have been circumferentially compressed at temperatures close to T_g_ of the particular terpolymer. Next, after fast cooling, the diameter of compressed scaffolds was *ca.* 7 mm (compression about 30%). Properties of the scaffolds with temporary shape have been presented in Table 2. As soon as possible, compressed scaffolds were sterilized with gamma radiation and electron beam, applying the following doses: 10, 15, and 25 kGy.

### 2.2. Influence of Gamma Irradiation on the Properties of Matrices and Scaffolds’ Morphology

Changes in properties of the polymeric matrix and scaffolds after absorption of γ irradiation doses are presented in Table 2 (where the numbers of scaffolds correspond to those shown in Table 1, with additional indication C—in a compressed temporary shape).

**Figure 1:**
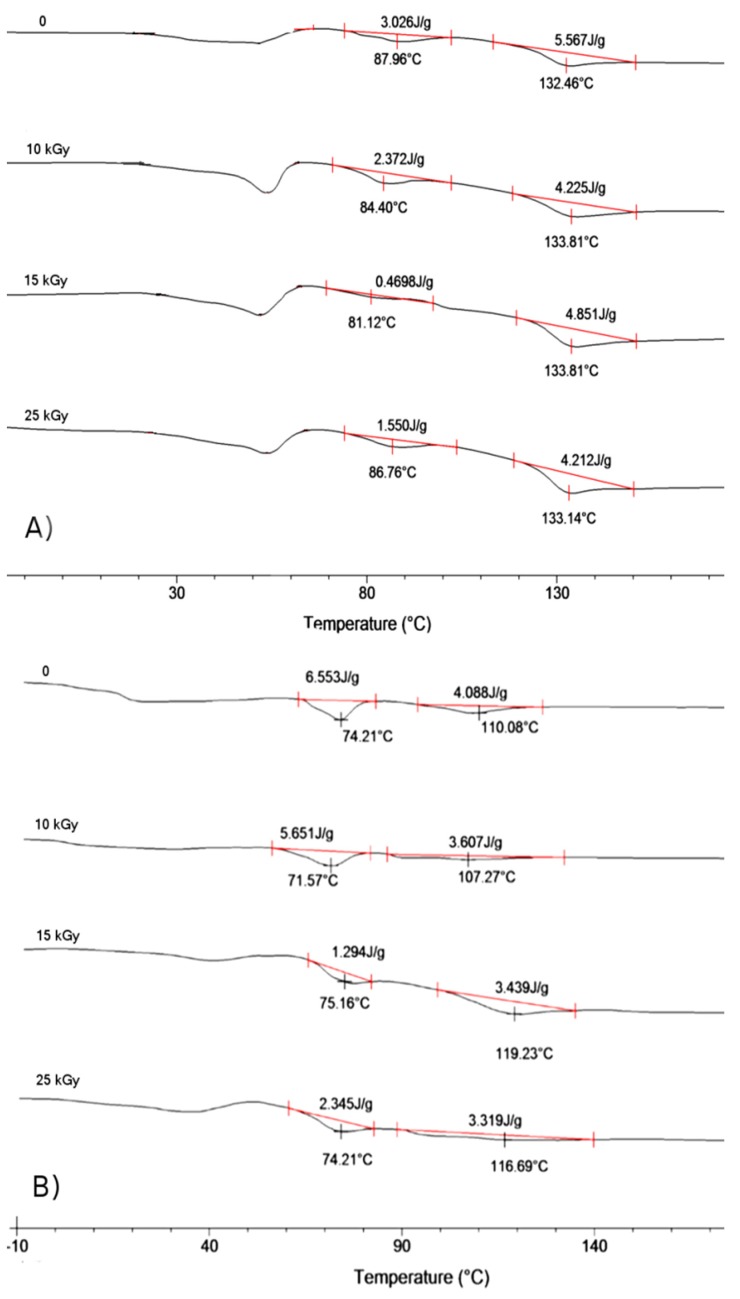

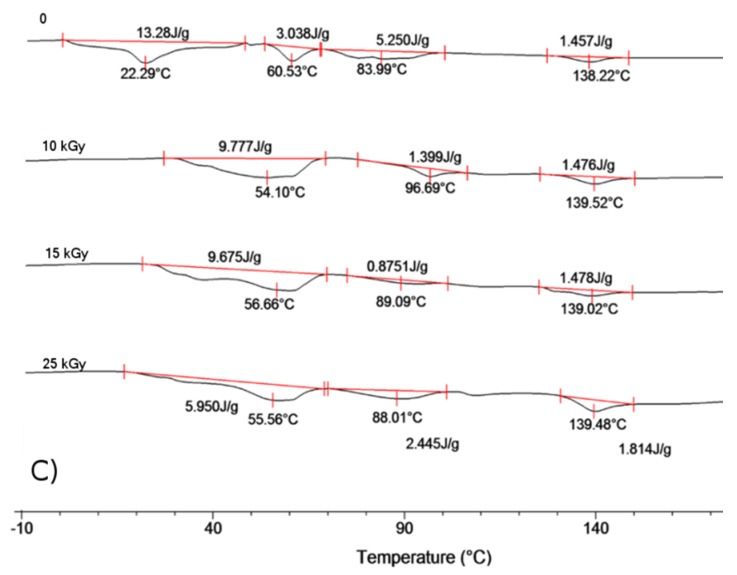
DSC traces for copolymer (**A**) LGT21 C; (**B**) LGT40 C; and (**C**) LTG C. I-heating run at 20°C/min after γ irradiation with dose: 0 kGy, 10 kGy, 15 kGy, and 25 kGy.

Gamma irradiation caused relatively strong degradation of copolymers’ chains resulting in a significant decrease in molecular weight and increase in molar mass dispersity. This effect was particularly noticeable for terpolymers containing carbonate units. In the case of terpolymer LGT40 relative molecular mass loss (dM_n_) exceeded 40% of initial average M_n_. Meanwhile, molecular weight of the sample LGT21, containing lower amounts of carbonate units, decreased up to 35% (Table 2). As a consequence, decreasing molecular weight together with a growing dose of irradiation resulted in a gradual drop in glass transition temperature. It is worth noting that DSC (differential scanning calorimetry) analysis has proved no changes in melting temperature of crystalline regions coming from the ordered phase of lactidyl microblocks in all tested terpolymers (Figure 1).

**Table 1:** Properties of investigated scaffolds and used polymeric matrices.

No.	Properties of Polymeric Matrix				Properties of Scaffold			
	Composition (mol. %)	M_n_ (kg/mol)	Đ	T_g_ (°C)	Average Porosity P (%)	Average Pore Diameter (µm)	Scaffold Diameter (mm)	Compressive Strength of Dried Scaffolds (MPa)
LGT21	l-lactide—68 Glycolide—11 TMC units—21	36.8	2.3	47	86 ± 5	395 ± 59	10.1 ± 0.2	0.8 ± 0.1
LGT40	l-lactide—46 Glycolide—14 TMC units40	23.9	1.9	28	88 ± 9	410 ± 67	10.0 ± 0.3	0.6 ± 0.1
LGC	l-lactide—79 Glycolide—10 Caprolactone—11	35.1	2.2	55	83 ± 3	380 ± 45	10.2 ± 0.3	0.9 ± 0.2

Notes: M_n_—average number molar mass determined with GPC; Đ—molar mass dispersity; T_g_—glass transition temperature determined with DSC (II run for amorphous sample obtained by quenching from melt). Presented data regarding scaffold properties were calculated as an average of five samples of each type of the scaffolds ± standard deviation (SD).

**Table 2:** Effect of γ irradiation dose on properties of terpolymers and scaffolds at their temporary shape.

No.	Properties of Polymeric Matrix					Properties of Scaffold at Temporary Shape		
	Dose (kGy)	M_n_ (kg/mol)	dM_n_ (%)	Đ	T_g_ (°C)	Average Porosity P (%)	Scaffold Diameter (mm)	Compressive Strength of Dried Scaffolds (MPa)
LGT21 C	0	36.8	-	2.3	47	55 ± 5	6.8 ± 0.3	1.1 ± 0.1
	10	32.7	11	2.4	46.6	58 ± 7	7.3 ± 0.4	1.1 ± 0.2
	15	29.8	19	2.5	46.4	57 ± 6	7.4 ± 0.3	1.0 ± 0.2
	25	23.6	36	2.7	45.6	58 ± 7	7.4 ± 0.4	0.7 ± 0.1
LGT40 C	0	23.9	-	1.9	28	58 ± 5	7.0 ± 0.4	0.6 ± 0.1
	10	19.4	19	2.5	26.7	61 ± 7	7.8 ± 0.5	0.4 ± 0.1
	15	17.1	29	2.5	26.5	62 ± 4	7.8 ± 0.3	0.4 ± 0.2
	25	13.2	45	2.8	25	61 ± 5	7.9 ± 0.4	0.2 ± 0.1
LGC C	0	35.1	-	2.2	55	53 ± 3	6.7 ± 0.3	0.9 ± 0.2
	10	31.7	10	2.5	53.6	57 ± 4	7.4 ± 0.3	0.8 ± 0.2
	15	29,8	15	2.9	53.5	58 ± 5	7.6 ± 0.5	0.8 ± 0.1
	25	26,2	25	2.9	52.6	58 ± 4	7.4 ± 0.4	0.5 ± 0.2

Notes: M_n_—average number molar mass determined with GPC; dM_n_—relative molecular mass loss; Đ—molar mass dispersity; T_g_—glass transition temperature determined with DSC (II heating run for amorphous sample obtained by quenching from melt). Presented data regarding scaffold properties were calculated as an average of five samples of each type of the scaffolds ± SD.

**Figure 2:**
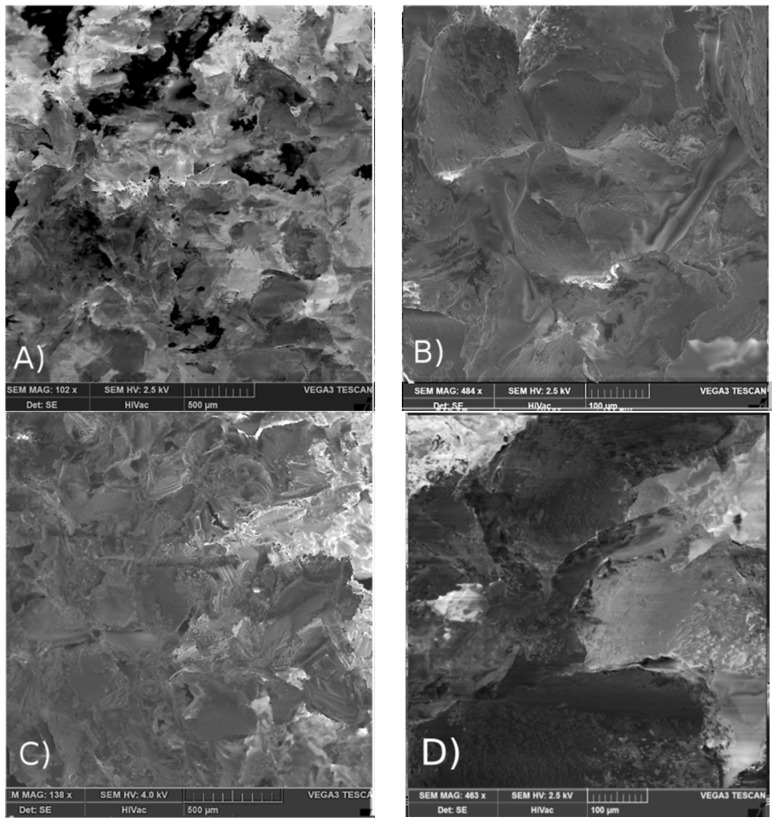
SEM pictures of porous surface of scaffold LGC C: (**A**,**B**) before sterilization; (**C**,**D**) after γ irradiation with a 25 kGy dose.

**Figure 3:**
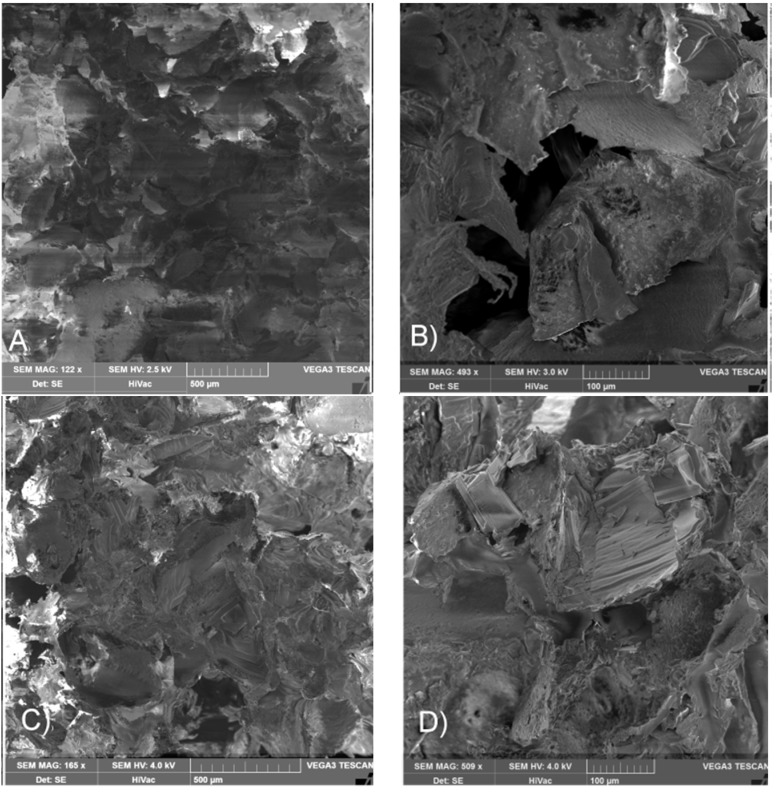
SEM pictures of porous surface of scaffold LGT40 C; (**A**,**B**) before sterilization and (**C**,**D**) after γ sterilization with irradiation dose of 25 kGy.

Growing destruction of terpolymers’ chains combined with an increasing level of absorbed irradiation reflects an essential decrease in mechanical properties of sterilized scaffolds (Table 2). The highest decrease in M_n_ denoted for scaffold LGT40 C, when treated with a 25 kGy dose, resulted in a decrease in compression strength of the material and reached only 1/3 of its initial value. In other cases, after treatment with the same dose of irradiation, the lowering mechanical properties were also significant. Scanning electron microscopy analysis of scaffolds’ surfaces after γ irradiation has revealed no changes in morphology of pore surface. Pictures demonstrating the highest degradation rate are presented in Figure 2 (sample LGC C) and Figure 3 (sample LGT40 C). Sterilization via gamma irradiation did not considerably affect scaffold morphology (Table 2). Dimensions of compressed samples remained practically unchanged.

### 2.3. Influence of Electron Beam Sterilization on the Properties of Matrices and Scaffolds Morphology

Changes in properties of polymeric matrix and scaffolds after treatment with various doses of electron beam are presented in Table 3.

**Table 3:** Effect of absorbed dose of electron beam on properties of terpolymers and scaffolds at their temporary shape.

No.	Properties of Polymeric Matrix					Properties of Scaffold at Temporary Shape		
	Dose (kGy)	M_n_ (kg/mol)	dM_n_ (%)	Đ	T_g_ (°C)	Average Porosity P (%)	Scaffold Diameter (mm)	Compressive Strength of Dried Scaffolds (MPa)
LGT21 C	0	36.8	-	2.3	47	55 ± 5	6.8 ± 0.3	1.1 ± 0.1
	10	34.1	7	2.4	47	57 ± 6	7.2 ± 0.5	1.1 ± 0.2
	15	32.8	11	2.4	46.3	56 ± 4	7.1 ± 0.3	1.0 ± 0.2
	25	25.8	30	3.2	45.8	57 ± 4	7.2 ± 0.4	0.9 ± 0.2
LGT40 C	0	23.9	-	1.9	28	58 ± 5	7.0 ± 0.4	0.6 ± 0.1
	10	22.0	8	2.0	25	57 ± 6	7.1 ± 0.5	0.6 ± 0.2
	15	20.2	15	2.0	25	60 ± 7	8.3 ± 0.5	0.7 ± 0.3
	25	19.2	20	2.3	24.5	63 ± 4	8.5 ± 0.5	0.5 ± 0.2
LGC C	0	35.1	-	2.2	55	53 ± 3	6.7 ± 0.3	0.9 ± 0.2
	10	33.7	4	2.2	53	54 ± 4	7.1 ± 0.2	0.9 ± 0.1
	15	33.1	6	2.3	53.5	59 ± 4	7.4 ± 0.3	0.9 ± 0.2
	25	30.8	12	2.7	52.6	60 ± 6	7.6 ± 0.4	0.7 ± 0.3

Notes: M_n_—average number molar mass determined with GPC; dM_n_—relative molecular mass loss; Đ—molar mass dispersity; T_g_—glass transition temperature determined with DSC (II heating run for amorphous sample obtained by quenching from melt). Presented data regarding scaffold properties were calculated as the average of five samples of each type of the scaffolds ± SD.

**Figure 4:**
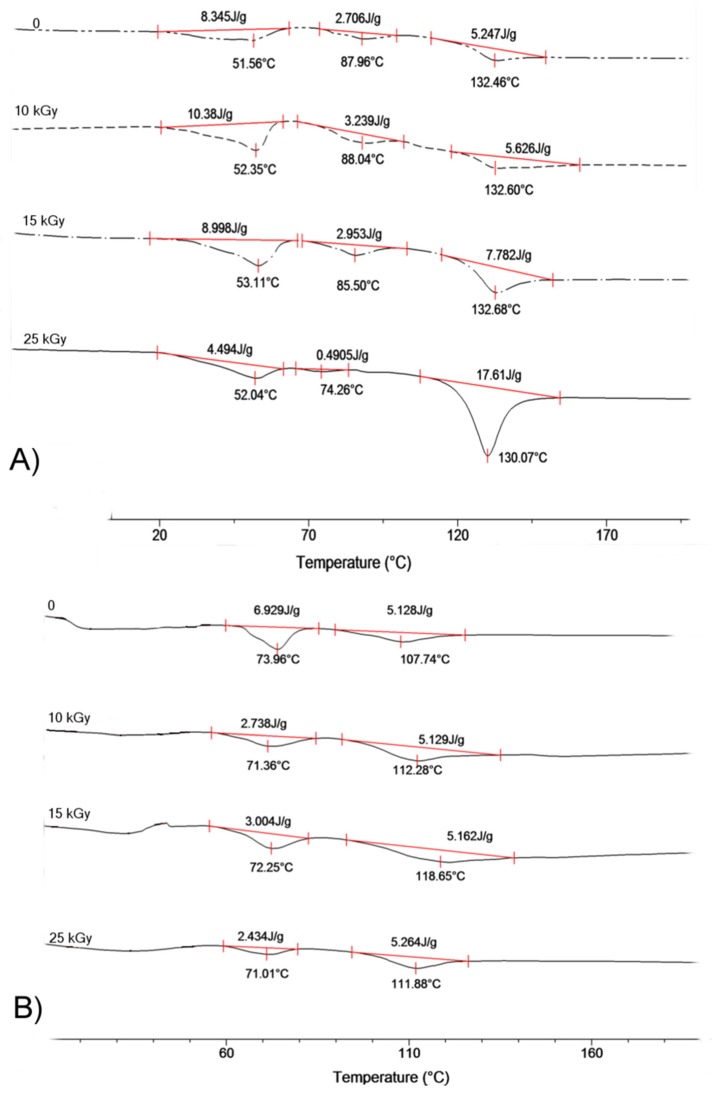

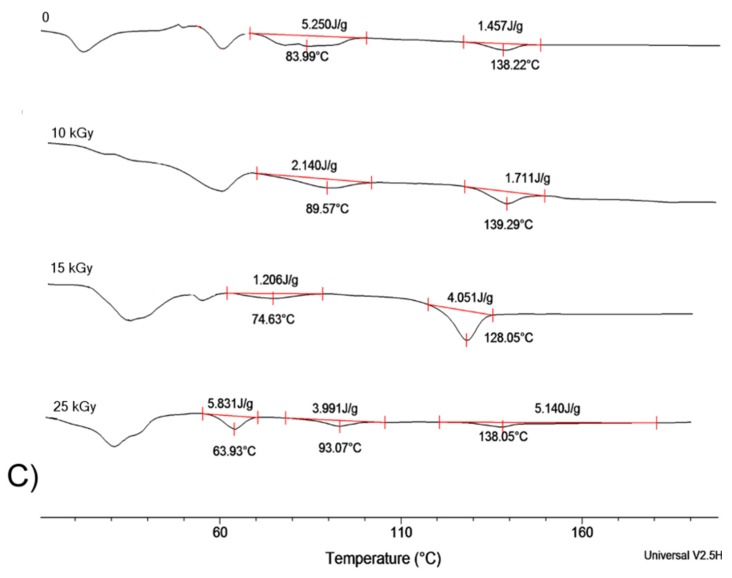
DSC trace for copolymer (**A**) LGT21 C; (**B**) LGT40 C; and (**C**) LGC C. I-heating run at 20 °C/min after electron beam irradiation with dose: 0 kGy, 10 kGy, 15 kGy, and 25 kGy.

Decrease in molecular weight of all the samples sterilized with electron beam were lower as compared to sterilization by gamma irradiation (Table 2 and Table 3; see also Supplementary Materials Figures S4–S6). The highest drop in M_n_ of the polymers has been noticed (similarly like in the case of γ irradiation) for terpolymers containing carbonate microblocks. Contrary to results obtained using γ irradiation, noticed in this case is a decrease in molecular weight which was similar for both scaffolds containing TMC units. In the case of sample LGT40, when treated with a 25 kGy dose, the decrease in M_n_ was about 20% as compared to initial molecular mass. This drop in M_n_ was half in comparison with the samples sterilized with γ irradiation. The decrease in molecular weight for sample LGT21 reached 30% of initial value. Relatively low changes of molecular weight (12% drop of M_n_) for scaffolds LGC C composed of LA/GL/CL units and, as a consequence, a noticeable decrease in their glass transition temperature has been observed (Figure 4).

Sterilization of polymeric matrices LGT21 C and LGT40 with electron beam at doses 15 kGy and even higher caused a slight increase in melting enthalpy (Figure 4). The most probable explanation of this phenomenon is the increase of matrix arrangement caused by growing mobility of terpolymer chains induced by the temperature rise of the material caused by the action of electrons. This phenomenon might have also affected the surface morphology of examined scaffolds. The influence of electron beam on the changes of surface properties of polylactide has been already reported [21]. In the presented study, this effect has much stronger impact on the samples, because prepared 3D scaffolds possess a unique, porous structure with very high interconnectivity, built from thin-walled biodegradable terpolymer.

**Figure 5:**
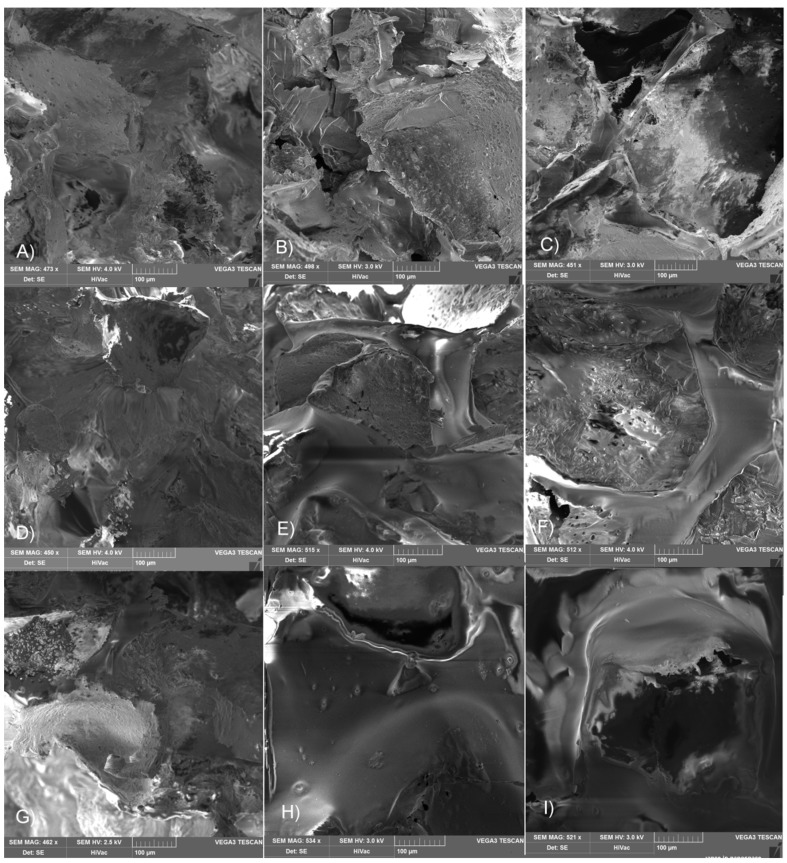
SEM pictures of scaffolds pore surface after electron beam sterilization made of terpolymer LGT21 C with dose (**A**) 10 kGy; (**B**) 15 kGy; (**C**) 25 kGy; terpolymer LGT40 C with dose (**D**) 10 kGy; (**E**) 15 kGy; (**F**) 25 kGy and terpolymer LGC C with dose (**G**) 10 kGy; (**H**) 15 kGy; and (**I**) 25 kGy.

Among SEM pictures presented in Figure 5, we can observe, that in the case of scaffold LGT40 C only samples irradiated with the lowest dose, present the morphology identical with initial ones. The dose of 15 kGy, and especially 25 kGy, caused significant changes in the surface of pores, resulting in melting of the polymeric matrix. This may be caused by the fact that this terpolymer exhibits the lowest glass transition temperature among all other samples (higher amount of TMC units as compared to sample LGT21 C).

The largest changes have been observed for scaffold LGC C (Figure 5H,I) made from terpolymer with the highest T_g_ (determined by DSC method). In spite of a high T_g_ value of this terpolymer, the observed “flowing” effect may be caused by the presence in the polymer chain caproil sequences resulting in an increase of chain mobility even in lower temperatures during heating, which comes from the growing level of absorbed electron energy (T_g_ is a temperature range rather, than a specific temperature).

### 2.4. Effect of Sterilization on Shape Memory Properties

Effect of sterilization process on the shape memory properties of the scaffolds (return time and extent of compressed samples) has been investigated by comparative study, in which results related to sterilized scaffolds were compared to non-irradiated ones (Table 4). The recovering temperatures were selected so as to initialize the return of shape, and at the same time were close to body temperature.

Although the sterilization effect at the 25 kGy irradiation dose has been noticed for all tested samples, in the majority of cases this impact was rather minor. No visible changes on the pore surface after returning to the temporary shape was observed (Supplementary Materials, Figure S7) and the shape recovery ratio was high. Scaffolds made with terpolymer LGT40 exhibiting the lowest glass transition temperature, in the case of gamma irradiation, demonstrated almost twice as slow are turn to permanent shape, and the extent of the return was much lower when compared to a reference sample. The main reason of this behavior was probably a very high decrease in molecular weight of the terpolymer, which is related to shortening chain length and destruction of its initial microstructure which is responsible for the shape memory effect (chain entanglement, phase separation, or presence of hydrogen bonds).

Scaffolds made from terpolymer LGT40 and sterilized with electron beam practically did not exhibit shape memory behavior. This was caused by their total relaxation as a consequence of the heating effect generated during the sterilization process. As a result of this process, directly after irradiation, a slight return of scaffold’s diameter from initial 7.7 to 8.5 mm has been observed.

**Table 4:** Influence of sterilization method on shape memory behavior of the scaffolds.

The Type of Sterilization	No.	Dose (kGy)	T_r_ (°C)	e_m_ (mm)	D_s_ (mm)	e_r_ (mm)	t_R_ (s)	R_r_ (%)
Unsterilized	LGT21C	0	37	10.1	6.8	9.7	600	96
	LGT40 C	0	30	10.0	7.0	9.6	320	96
	LGC C	0	41	10.2	6.7	9.4	340	94
Gamma Irradiation	LGT21 C	25	37	10.1	7.4	9.1	690	89
	LGT40 C	25	30	10.0	7.9	8.6	520	86
	LGC C	25	41	10.2	7.4	9.2	380	90
Electron Beam	LGT21 C	25	37	10.1	7.2	9.6	620	95
	LTG40 C	25	30	10.0	8.5	8.5	>1000	^nd^
	LGC C	25	41	10.2	7.6	9.1	410	89

Notes: No.—type of the scaffold according to Table 2; T_r_—recovering temperature, e_m_—diameter of sample before compression; D_s_—diameter sample after sterilization; e_r_—diameter of sample after recovery; t_R_—shape recovery time; R_r_—maximal shape recovery ratio (Equation (1)). Presented data were calculated as an average of three samples of each type of the scaffolds. ^nd^—not determined.

## 3. Experimental Section

### 3.1. Terpolymers Synthesis and Characterization

#### 3.1.1. Materials and Chemicals

l-Lactide, glycolide (Glaco Ltd., Beijing, China) were purified by recrystallization from ethyl acetate solution and dried in a vacuum oven at room temperature; ε-caprolactone (Fluka, Steinheim, Germany) was dried with calcium hydride and distilled under argon atmosphere before use. Trimethylene carbonate (Ingelheim Boehringer, Ingelheim am Rhein, Germany), dry chloroform and methanol (Aldrich, Steinheim, Germany) were used as received. Initiators and catalysts: zirconium (IV) acetylacetonate—(Zr(acac)_4_), zinc(II) acetylacetonate monohydrate, 1,4-butanediol (Zn(acac)_2_xH_2_O), (Aldrich, Steinheim, Germany) were used as received.

#### 3.1.2. Synthesis of l-lactide/glycolide/trimethylene Carbonate Terpolymers

The first stage to produce terpolymers containing carbonate units was the synthesis of hydroxyl-terminated oligo(trimethylene carbonate), which were obtained by ROP of TMC. The oligomerization was conducted in bulk, at 120 °C with presence of 1,4-butandiol as initiator and Zn(acac)_2_x H_2_O as a catalyst with catalyst/monomer ratio (C/M) of 1:3000 (Scheme 1). After 2 h monomer conversion more than 99% was obtained. The detailed course of the oligomerization was described previously [8,18]. Obtained oligotrimethylene carbonate, with average number molecular mass M_n_ = 5100 g/mol, molecular mass dispersity Đ = 2.1(both results determined by GPC) was used as a macroinitiatorin copolymerization of l-lactide with glycolide (with lactide/glycolide/initiator molar ratio as; 70/10/0.4 for LTG21 or 55/10/0.9 for LTG40). This reaction was also conducted in bulk at 120 °C with the use Zr(acac)_4_ as a catalyst with C/M molar ratio as 1:2500. After 12 h, a terpolymer LTG21 was obtained with a yield of about 98%, M_n_ = 36 800 g/mol and LTG40 with yield 91%, M_n_ = 23,900 g/mol. This product were dissolved in chloroform, precipitated with cold methanol, and then vacuum-dried to a constant weight. Examples of obtained chromatograms (Figures S4 and S5) and NMR spectra (Figures S1 and S2) are published in Supplementary Materials.

#### 3.1.3. Synthesis of l-lactide/glycolide/ε-caprolactone Terpolymer

Terpolymerization of glycolide with l-lactide and ε-caprolactone was performed in bulk at 100 °C, in one pot ROP reaction with Zr(acac)_4_ as an initiator and initiator/monomers molar ratio I/M as 1: 1000 (Scheme 2) according to previously described methods [19]. After 48 h, terpolymer LTC was obtained with a yield of about 96%.This product was dissolved in chloroform, precipitated with cold methanol, and then vacuum-dried to a constant weight. An example of the obtained chromatogram (Figure S5) and NMR spectrum (Figure S3) are published in Supplementary Materials.

#### 3.1.4. Terpolymers and Scaffolds Characterization

Average number molecular weights (M_n_) and molar mass dispersity (Đ) of the synthesized terpolymer samples were measured using gel permeation chromatography (GPC) (Viscotek apparatus Rimax, Malvern Instruments, Worcestershire, England, chloroform, temperature 35 °C, flow 1 mL/min, using two Viscotek 3580 columns, refractive detector, calibration with polystyrene standards—Easi Vial PS-M calibration kit (Agilent, Santa Clara, CA, USA), with M_p_ from 1000 to 500,000 g/mol).

The composition and microstructure of the terpolymers’ chains were determined with NMR measurements. The NMR spectra of the terpolymers were recorded at 600 MHz with the Avance II Bruker Ultrashield Plus Spectrometer (Bruker Corporation, Anaheim, CA, USA) and a 5 mm sample tube. Dried deuterated chloroform was used as solvent and tetramethylsilane was used as the internal standard. The ^1^H NMR spectra were obtained with 32 scans, a 2.65 s acquisition time, and an 11 µs pulse width. The ^13^C NMR spectra were recorded at 150 MHz with the same spectrometer and conditions as the proton spectra. The acquisition time was 0.9 s, the pulse width was 9.4 µs, the delay between pulses was 2 s, and the spectral width was 36,000 Hz. During the conducted analysis of the terpolymer chain structure, the signals assignment previously published by us was used [8,19,22].

Thermal properties, such as glass transition temperature and heat of melting, were examined by DSC with a DuPont 1090B apparatus calibrated with gallium and indium (heating and cooling rate of 20 °C/min in the range from −100 to 220 °C, DuPont, Newark, DE, USA) according to the ASTM E 1356-08 standard [23].

Compressive strength and elastic modulus of the scaffolds were determined under compression using the Instron 4200 tensile testing machine (Illinois Tool Works, Glenview, IL, USA) with a ramp speed of 0.02 cm/s, at room temperature.

### 3.2. Formulation of Scaffolds and Methods of Their Characterization

The scaffold preparation procedure was described in detail elsewhere [24]. In brief, the scaffolds were produced by the classical solvent casting/particulate leaching technique. Sieved sodium chloride particles (POCh, Gliwice, Poland) of about 300–480 µm, were mixed with 10% (*w*/*v*) terpolymer solution in methylene chloride (Aldrich, Steinheim, Germany) to end up with a porosity of 85% after salt leaching. The mixture was transferred into a special Teflon form (diameter of mold cavity 10 mm and depth 8 mm) and quickly cooled down in the freezer to −80 °C. Then, the samples were lyophilized. The solid salt/polymer composites in the barrel forms were transferred into 1000 mL glass container ﬁlled with ultra-high quality water (UHQ-water, produced in UHQ PS system, Purelab, ELGA LabWater, Buckinghamshire. England). To enhance salt leaching, the water flow was assured by using of a magnetic stirrer operating at a speed of 50 rpm. The washing was stopped until the conductivity of the water was about 2 µS/cm, which usually took 4–5 days. The samples were dried in air and vacuum.

For scaffold structure characterization, thin slices were cut from a representative sections of the scaffolds, and images were captured on a scanning electron microscope (SEM, model Tescan VEGA 3SBU, Tescan Orsay Holding, Brno, Czech Republic). Accelerating voltage range was 1–3 kV to minimize the impact of beam energy on the observed samples (the samples were not coated with conductive layer that may have an impact on the morphology of the surface), and then observed under magnifications of about ×100 and ×500*.* All SEM images were performed at 23 °C under high vacuum, using secondary electron mode.

The scaffolds’ porosity was calculated from the density of the solid copolymer, the mass, and the dimensions of each scaffold, according to the ASTM standard D-3574-08 [25].

### 3.3. Deformation Scaffolds to Temporary Shape

Temporary shape formation of the scaffolds was done on a device designed for the experiment purpose by adaptation of self-centering four-jaw chuck. The central place where the scaffold was fixed was heated by infrared lamp for maintaining the required temperature. The device allowed for precise compression of small polymeric samples simultaneously in three directions due to regulation of jaws by screwing or unscrewing. After deformation process, fixed in the device sample was immediately cooled with compressed air, receiving in this way temporary shape. Such received samples, to avoid self-return to permanent shape caused by their relaxation, have been kept in a freezer at −60 °C prior to use.

### 3.4. Measurements of Scaffolds' Shape Memory Behavior

The free-strain recovery test was conducted under isothermal conditions, in water. The samples of scaffolds were heated to the required temperature rapidly by immersing in the water bath set at 20 °C, or 37 °C, or 40 °C and remained at that temperature measuring the recovered changes in length as a function of time.

The ability of the sample to recover was quantified by the shape recovery ratio parameter (R_r_ ), defined as:

R_r_ = (e_r_/e_m_) × 100% (1)

where, e_m_ is the final diameter of tested sample with temporary shape before sterilization; and e_r_ is the measured residual diameter after sterilization and one complete cycle of the shape memory experiment.

### 3.5. Conditions of Sterilization

The scaffolds, packed in argon atmosphere into rigid and tightly closed polystyrene packaging were transported in thermostatic containers at temperature below 8 °C to the Institute of Nuclear Chemistry and Technology in Warsaw for sterilization. The irradiation of the scaffolds was performed in a Gamma Chamber 5000 apparatus (BRIT, Navi Mumbai, India), using isotopes of ^6^°Co as a gamma radiation emitter at room temperature. A mean dose rate was 5.59 kGy/h. Sterilization using electron beams was conducted by means of the electron accelerator Elektronika 10/10 (Institute of Nuclear Chemistry and Technology, Warsaw, Poland) generating a beam with energy of 10 MeV and average power of 10 kW at room temperature too.

## 4. Conclusions

During designing and formation of scaffolds for cells growth purposes, one of the most important factors to be taken into consideration is choice of optimal, effective method of sterilization which does not, or eventually causes at a minimal level, undesirable side effects. In the case of materials exhibiting shape memory behavior induced thermally, it can be particularly difficult, considering that the temporary shape of the scaffolds must be kept after sterilization. With this respect, radiation sterilization seems to be the most proper method for these purposes.

Obtained results have proved that by keeping appropriate storage and transport conditions of scaffolds with temporary shape it is possible to sterilize them and maintain their designed shape. Generally, if the dose is not more than 10 kGy, sterilization with use of both methods, gamma radiation and electron beam, will not considerably affect the shape memory behavior. In the case of sterilized scaffolds prepared from terpolymers with relatively high glass transition temperature (above 40 °C), parameters describing the rate and extent of the return from temporary to permanent shape, even at high dose of irradiation, were comparable to non-treated samples.

Problems with maintaining shape memory behavior appeared only in the case when scaffolds prepared from terpolymer with lower T_g_ were irradiated by means of electron beam. For this type of scaffold, their ability to return to permanent shape was lost as a result of partial self-decompression and relaxation of the samples during sterilization. The reason of this phenomenon is heat generated as a result of conversion of the kinetic energy of the electrons. This process at doses of 15 kGy and higher caused essential changes in morphology of the pores’ surface.

Nevertheless, it should be kept in mind that the methods of sterilization proposed in the presented study (especially gamma irradiation) also possess few drawbacks, including strong degradation of polymer and drop in T_g_, resulting in a decrease in the mechanical properties of the scaffolds. By contrast, one of the advantages of γ-radiation use is the fact that it does not cause any noticeable changes of the pores’ surface, even at the highest dose of irradiation. For comparison, sterilization of the scaffolds by means of electrons beam caused much less negative influence on mechanical properties of treated samples. However, from the other side, using this type of sterilization, at the dose of 15 kGy, the effect of slight melting of pores’ surface of terpolymers containing caproil segments and high amount of carbonate units was observed.

Based on the results obtained, it can be concluded that the most optimal method for sterilization of porous, 3D scaffolds was electron beam irradiation at the dose not higher than 10 kGy. The higher dose of irradiation induces the higher, undesirable changes in the structure of scaffolds and as a consequence, decline in their shape behavior properties. It is also worth remembering that the sterilization is intended to destroy pathogens; however, their residues containing antigens, foreign proteins which are not removed during this treatment. With this respect, it is crucial to keep rules of good manufacturing practice during synthesis of terpolymers and preparation of the scaffolds afterward in order to limit microbiological contamination as much as possible.

Further investigations related to the possibilities of application presented in this study, such as scaffolds designed for large bone defect treatment, is being planned. Using the same procedure of scaffold preparation and methods of their sterilization (not exceeding a dose of 10 kGy), we would like to examine whether the sterility at the sterility assurance level (SAL) of 10^−6^ is possible. It is worth mentioning that, as presented in this study, scaffolds sterilized with electron beam at 10 kGy dose were successfully applied for evaluation of their cytotoxicity and shape memory properties in *in vitro* conditions [17].

## Supplementary Materials

The following are available online at www.mdpi.com/1996-1944/9/1/64/s1.
