# Supplementary material for: Influence of Radiation Sterilization on Properties of Biodegradable Lactide/Glycolide/Trimethylene Carbonate and Lactide/Glycolide/ε-caprolactone Porous Scaffolds with Shape Memory Behavior
Source: Materials (Basel). 2016 Jan 20;9(1):64. doi: 10.3390/ma9010064 (PMC5456516; doi:10.3390/ma9010064)
Supplement: Supplementary file 1 [file materials-09-00064-s001.pdf]

# Supplementary Materials: Influence of Radiation Sterilization on Properties of Biodegradable Lactide/Glycolide/Trimethylene Carbonate and Lactide/Glycolide/ε-Caprolactone Porous Scaffolds with Shape Memory Behaviour

**Piotr Rychter, Natalia Śmigiel-Gac, Elżbieta Pamuła, Anna Smola-Dmochowska, Henryk Janeczek, Wojciech Prochwicz and Piotr Dobrzyński**

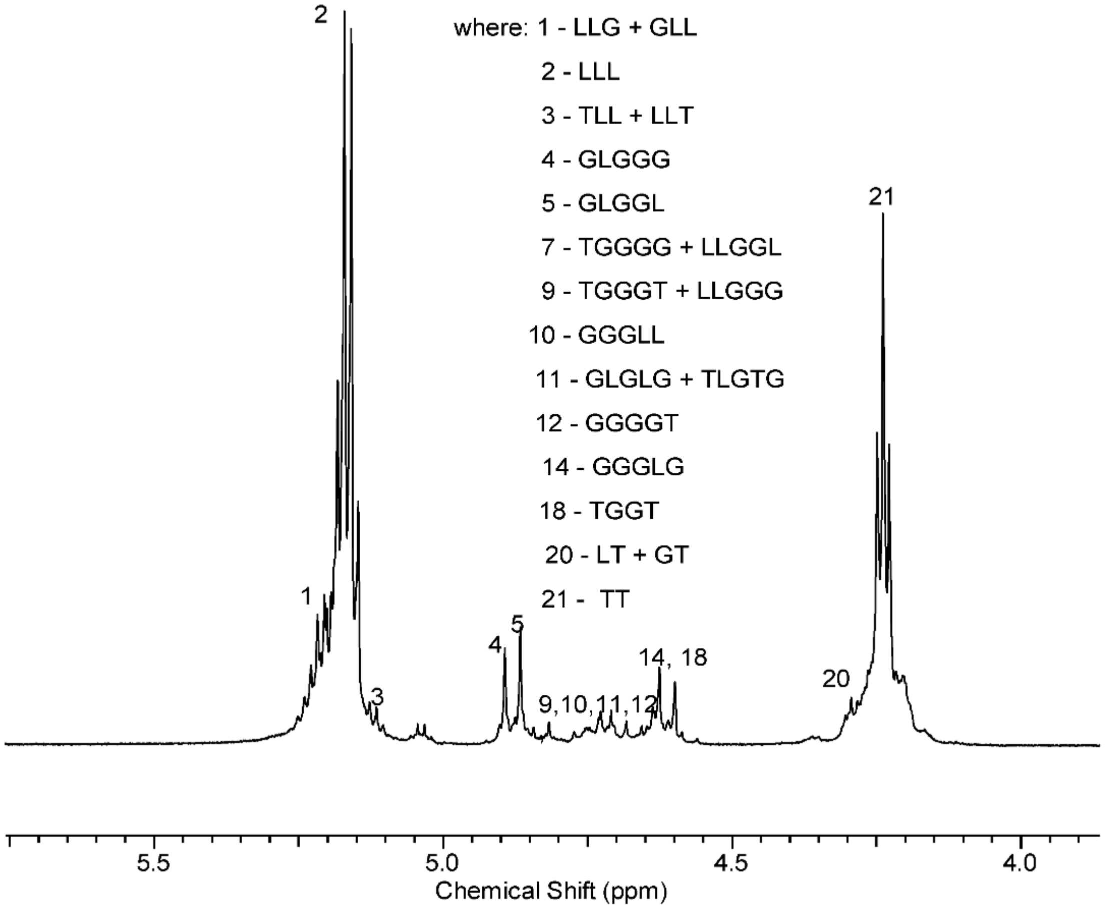


**Figure S1.** $^{1}$H NMR spectrum of terpolymer LGT21 (in $CDCl_3$).

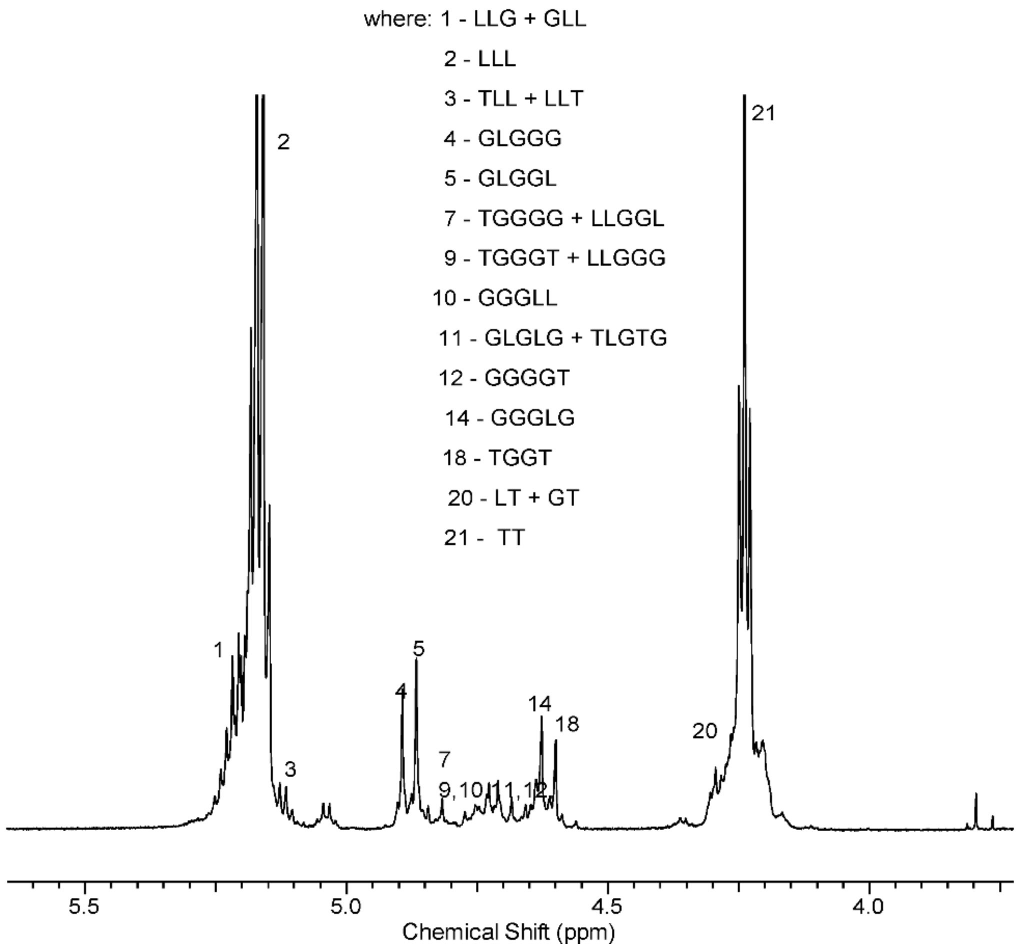


**Figure S2.** $^{1}H$ NMR spectrum of terpolymer LGT40 (in $CDCl_3$).

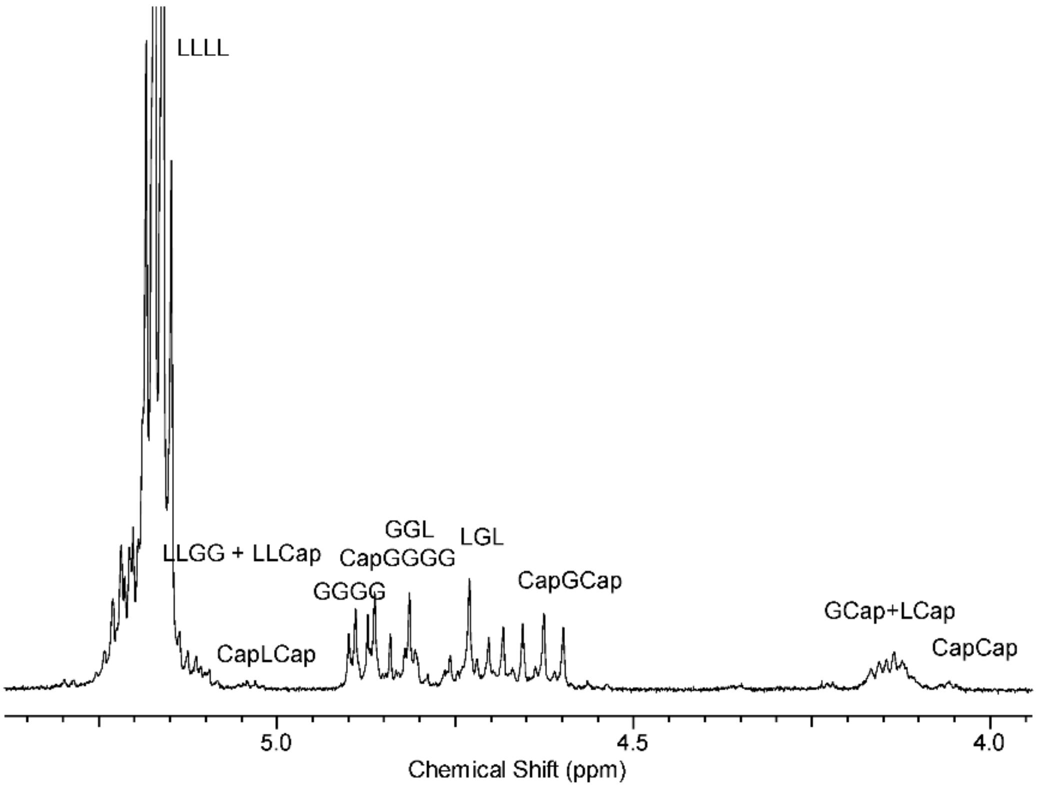


**Figure S3.** $^{1}H$ NMR spectrum of terpolymer LGC (in $CDCl_3$).

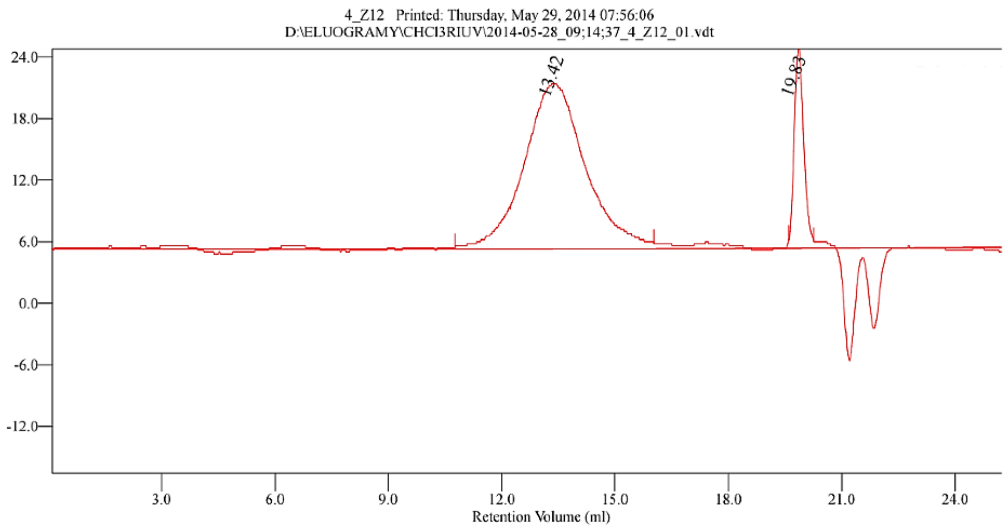


**A)**

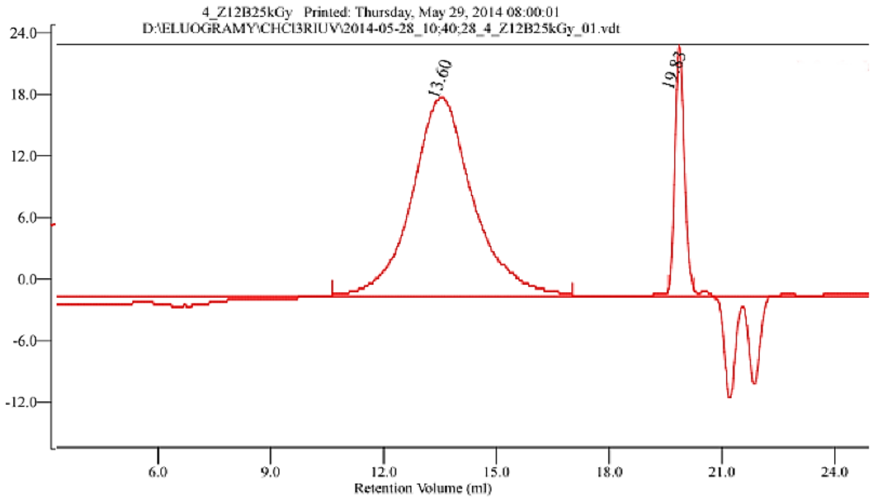


**B)**

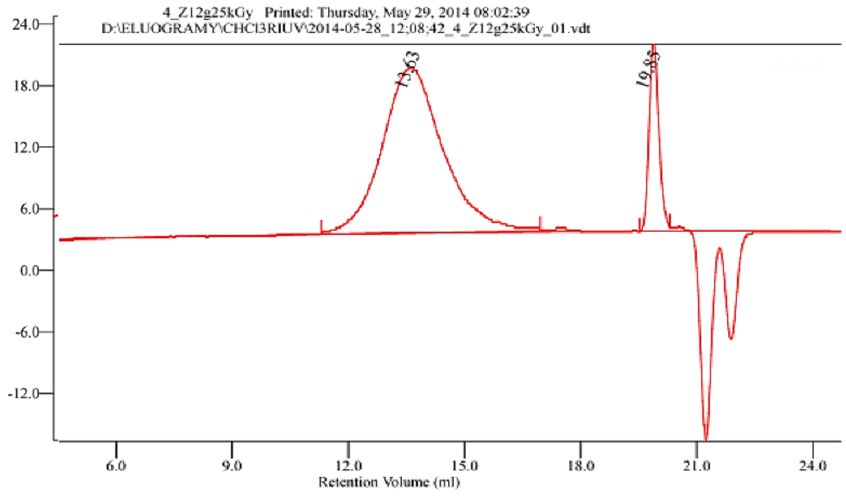


**C)**

**Figure S4.** GPC chromatograms for sample LGT21; (**A**) before irradiation; (**B**) after electron beam irradiation with 25 kGy dose; and (**C**) after γ irradiation with 25 kGy dose.

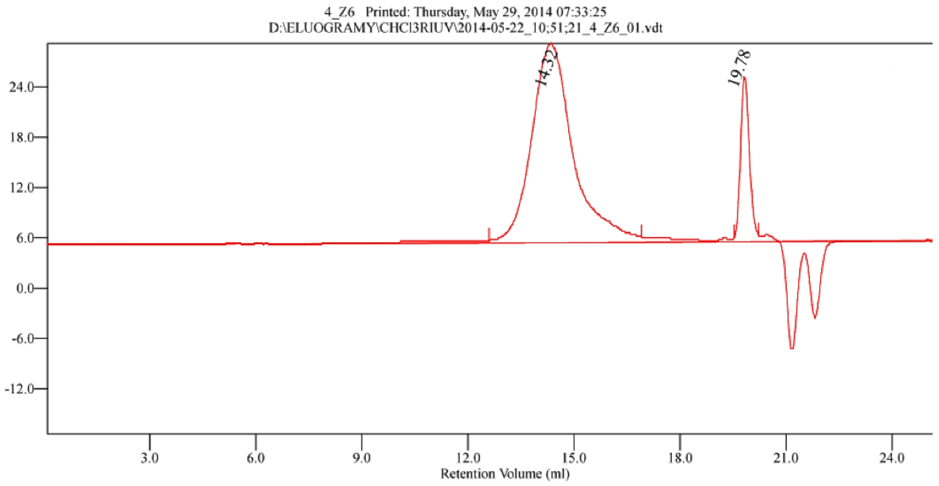


**A)**

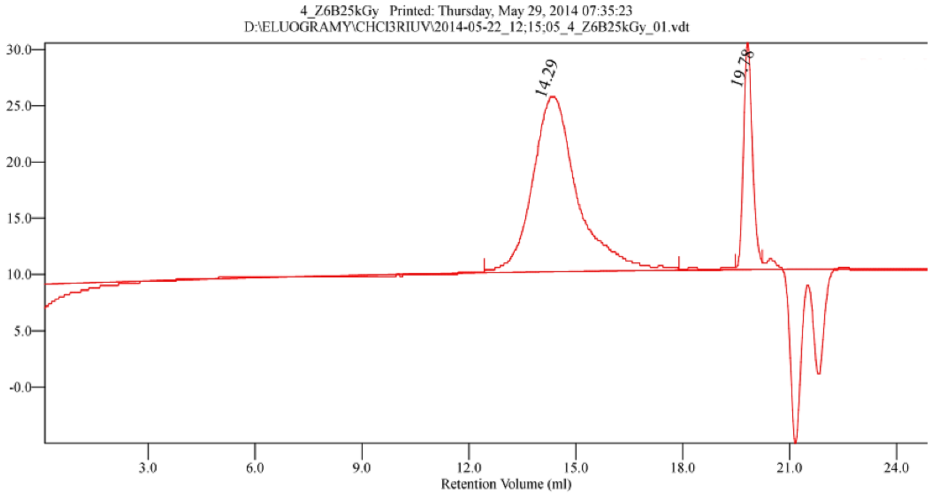


**B)**

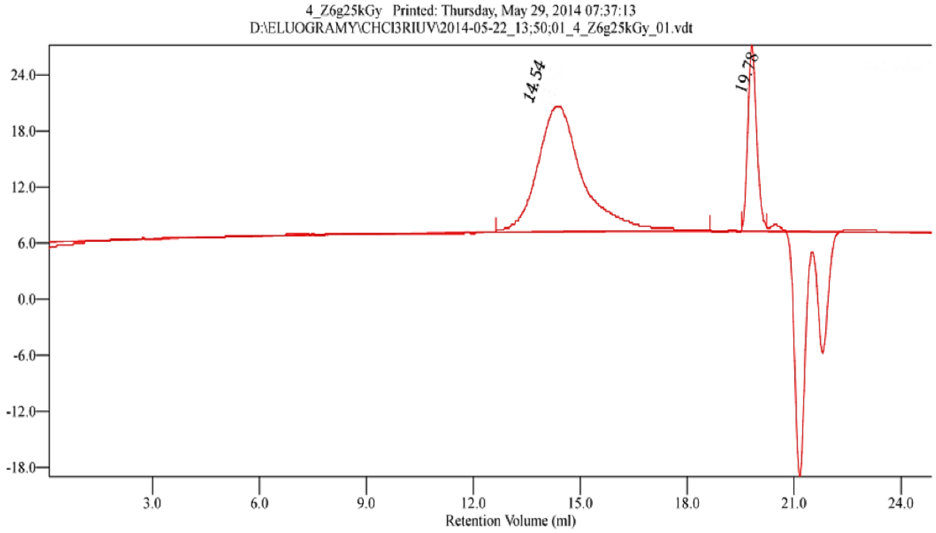


**C)**

**Figure S5.** GPC chromatogram for sample LGT40; (**A**) before irradiation; (**B**) after electron beam irradiation with 25 kGy dose; and (**C**) after $\gamma$ irradiation with 25 kGy dose.

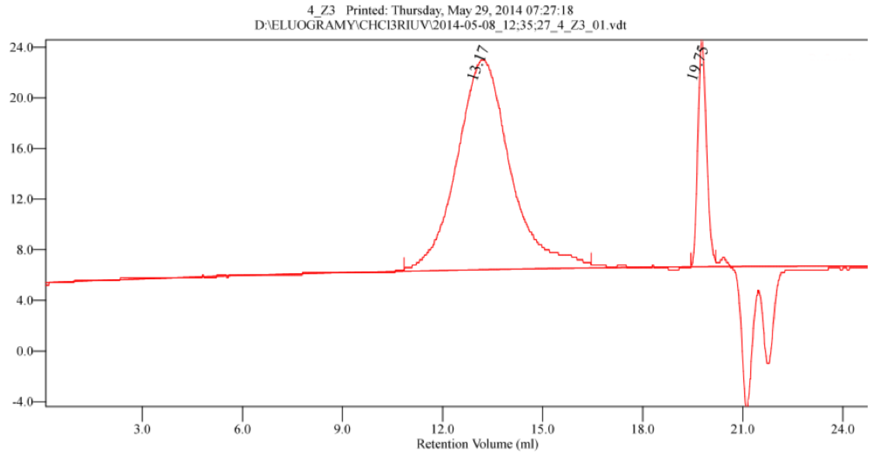


**A)**

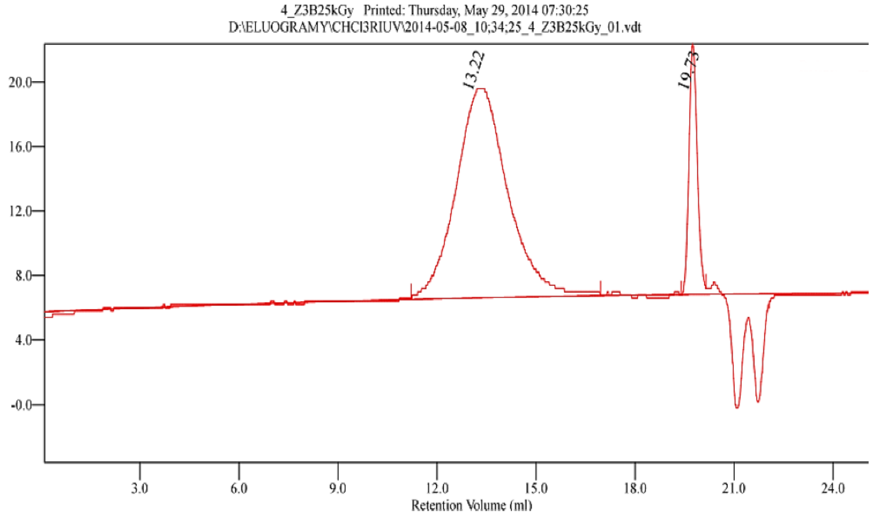


**B)**

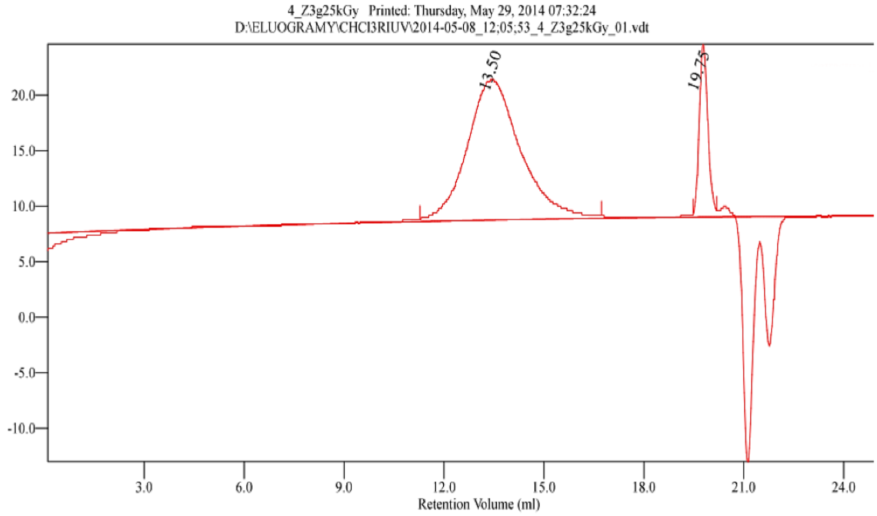


**C)**

**Figure S6.** GPC chromatograms for sample LGC; (**A**) before irradiation; (**B**) after electron beam irradiation with 25 kGy dose; and (**C**) after γ irradiation with 25 kGy dose.

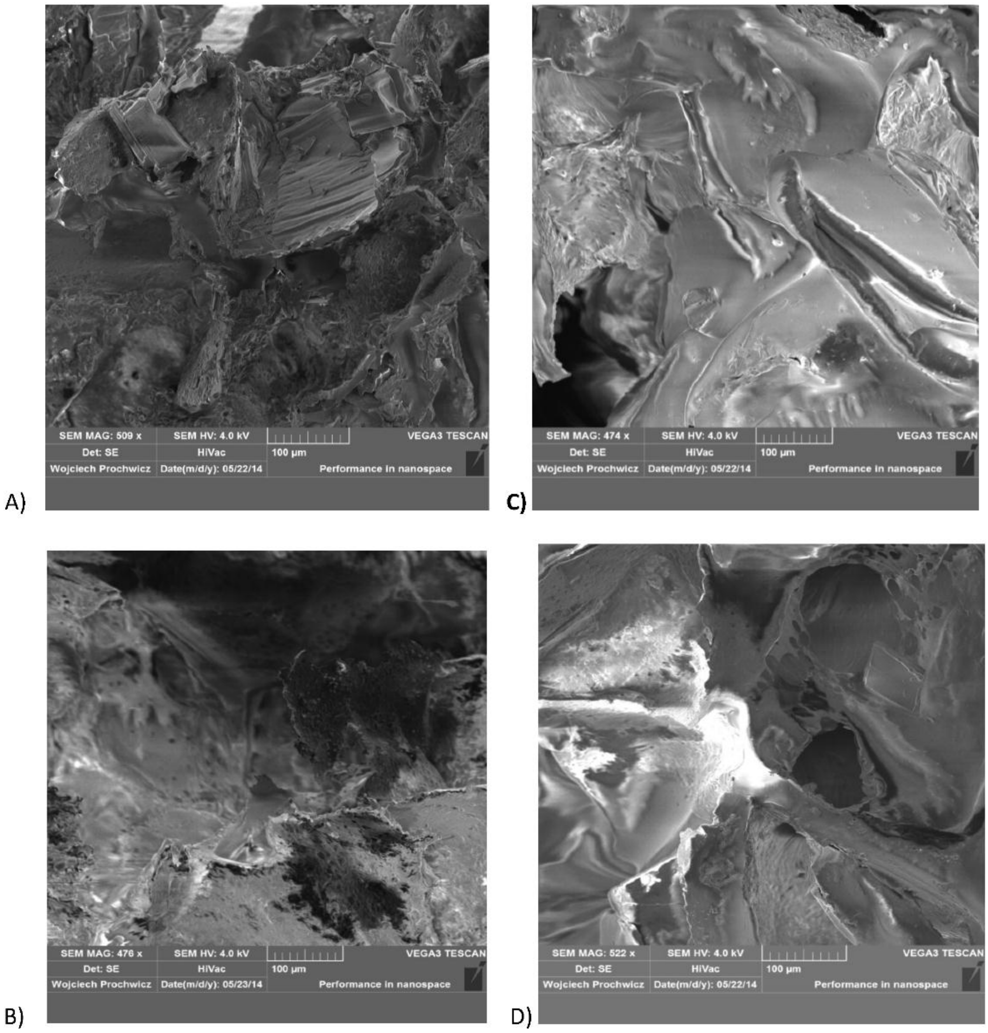


**Figure S7.** SEM pictures of porous surface of scaffolds; (**A**) LGT40 C scaffold after $\gamma$ sterilization with irradiation dose 25 kGy, in compressed temporary shape; (**B**) LGT40 C scaffold after $\gamma$ sterilization with irradiation dose 25 kGy, after recovery to permanent shape; (**C**) LGC C scaffold after electron beam sterilization with dose 25 kGy, in compressed temporary shape; and (**D**) LGC C scaffold after electron beam sterilization with dose 25 kGy, after recovery to permanent shape.
